# Helile formula from “Tai ping sheng hui fang*”*: anti-*Helicobacter pylori* activity, gut microbiota modulation, and inflammatory response regulation

**DOI:** 10.3389/fcimb.2025.1603128

**Published:** 2025-07-22

**Authors:** Ling Ou, Meiyun Chen, Chang Peng, Haobo Chen, Yajie Hao, Qingchang Chen, Zhong Feng, Meicun Yao, Xianhe Kong

**Affiliations:** ^1^ School of Pharmaceutical Sciences (Shenzhen), Sun Yat-sen University, Shenzhen, Guangdong, China; ^2^ Discipline of Chinese and Western Integrative Medicine, Jiangxi University of Chinese Medicine, Nanchang, China; ^3^ International Pharmaceutical Engineering Lab of Shandong Province, Feixian, Shandong, China; ^4^ Department of Biomedical Engineering, College of Design and Engineering, National University of Singapore, Singapore, Singapore; ^5^ Nanchang Research Institute, Sun Yat-sen University, Jiangxi, China; ^6^ Clinical Medical Research Center for Plateau Gastroenterological Disease of Xizang Autonomous Region, Affiliated Hospital of Xizang Minzu University, Xianyang, Shanxi, China; ^7^ Department of Gastrointestinal Endoscopy, The Sixth Affiliated Hospital, Sun Yat-sen University, Guangzhou, China; ^8^ Biomedical Innovation Center, The Sixth Affiliated Hospital, Sun Yat-sen University, Guangzhou, China

**Keywords:** helile formula, *Helicobacter pylori*, material basis, antibacterial activity, mechanism

## Abstract

**Background:**

*Helicobacter pylori* (HP) is a major gastric pathogen linked to chronic gastritis, peptic ulcers, and gastric cancer. The emergence of antibiotic resistance has prompted the search for alternative treatments. Helile formula, derived from the ancient “Taiping Shenghuifang,” is known to treat various degrees of diarrhea and has potential for treating gastrointestinal disorders. However, the antibacterial efficacy, material basis, and action mechanisms of the helile formula against HP remain undetermined.

**Methods:**

The chemical constituents were analyzed using ultra-high-performance liquid chromatography-tandem mass spectrometry (UPLC-MS/MS) and high-performance liquid chromatography (HPLC). The *in vitro* anti-HP activity and underlying mechanisms were investigated through a series of assays, including the determination of minimum inhibitory concentration (MIC), N-phenyl-1-naphthylamine (NPN) uptake assay, scanning electron microscopy (SEM) to observe morphological changes, cell viability and cell adhesion activity assays, assessment of nitric oxide (NO) production, and reverse transcription-quantitative polymerase chain reaction (RT-qPCR) for gene expression analysis. For the *in vivo* anti-HP infection study and mechanism exploration, techniques such as hematoxylin and eosin (H&E) staining for histological examination, enzyme-linked immunosorbent assay (ELISA) for cytokine and antibody quantification, 16S ribosomal DNA (16S rDNA) sequencing for microbial community profiling, and metabolomics for global metabolite analysis were employed.

**Results:**

Multiple constituents of helile formula, namely ellagic acid, gallic acid, chebulagic acid, chebulic acid, and corilagin, were identified. *In vitro*, helile formula increased bacterial outer-membrane permeability, disrupted HP structure, inhibited toxin-related genes, and suppressed cell adhesion. In male Kunming mice, helile formula effectively reversed HP-induced inflammation. It modulated key metabolites, such as adenine, panaxytriol, 4-hydroxyglutamate semialdehyde, and N-alpha-Acetyl-L-lysine. It influenced the gut microbiota, especially families like *Muribaculaceae* and *Lactobacillaceae*. Adenine, in particular, repaired HP-caused damage to GES1 cells, reduced HP - mediated cell adhesion, and inhibited HP-induced interleukin-6 (IL-6) and tumor necrosis factor-alpha (TNF-α) production.

**Conclusion:**

These findings demonstrated the remarkable anti-HP efficacy of the helile formula *in vitro* and *in vivo*, suggesting that the helile formula represents a highly promising therapeutic candidate for the management of HP infections.

## Graphical Abstract

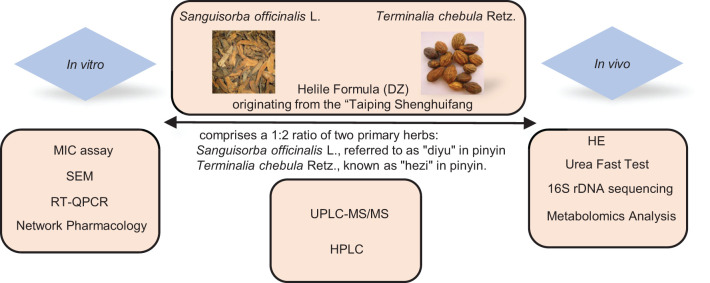

## Introduction

*Helicobacter pylori* (HP) is a prevalent pathogenic bacterium that can persist in the human gastrointestinal tract for extended periods, posing risks to human health (Kamangar et al., 2011). Extensive research indicates that HP is a major cause of gastritis, leading to inflammation of the gastrointestinal tract and gastric wall degeneration, resulting in symptoms such as stomach pain, vomiting, and diarrhea (Marshall and Warren, 1984; Kamangar et al., 2011; Myint et al., 2015). Additionally, studies have found that HP can disrupt the protective barrier of the gastric wall, causing gastric acid to ulcerate the stomach lining and epithelial damage, thereby leading to gastric ulcers (Chan et al., 1991; Robinson et al., 2017). Chronic HP infection can induce persistent inflammation in the gastrointestinal tract, potentially leading to malignancies in the gastrointestinal system.

Treatment options for HP infection typically involve a combination of antibiotics and acid-suppressing medications. The most common treatment regimen is known as triple therapy, which includes two different antibiotics (such as clarithromycin, amoxicillin, or metronidazole) along with a proton pump inhibitor to reduce stomach acid production, which is usually taken for a period of 10 to 14 days (Chey et al., 2017; Malfertheiner et al., 2017). However, this treatment is accompanied by a range of side effects. Patients often experience gastrointestinal disturbances such as nausea, vomiting, diarrhea, abdominal discomfort, and constipation. Allergic individuals may develop skin itching, rashes, and facial or limb swelling. Additionally, the overuse of antibiotics has led to an alarming increase in antibiotic-resistant HP and other bacterial strains (Fauzia and Tuan, 2024). Prolonged antibiotic use also disrupts the gut microbiota, exacerbating gastrointestinal problems, and can lead to other symptoms like headaches, fatigue, muscular aches, dizziness, and sleep disruptions. Once resistance develops, treatment duration is prolonged, stronger or alternative antibiotics may be required, treatment costs increase and treatment failure becomes more likely, severely threatening patients’ health (Setshedi and Smith, 2023).

In light of these challenges, there is a growing interest in alternative therapies, particularly natural remedies and herbal alternatives. Research has indeed demonstrated the potential of natural remedies and herbal alternatives in addressing HP infections (Zhong et al., 2022). For instance, compounds like berberine, epiberberine, and palmatine play a role in mitigating the inflammation and tissue damage caused by HP infections (Chen et al., 2020). Over the recent years, our research team has made substantial advancements in investigating the therapeutic potential of traditional Chinese medicine (TCM) against HP. We have conducted extensive research in the treasure trove of Chinese herbal remedies, uncovering and validating a series of plants with potent anti-HP activity *in vitro*. Key herbs such as *Sanguisorba officinalis* L (Shen et al., 2021)., *Syzygium aromaticum* (Peng et al., 2022), and *Terminalia chebula* Retz (Ou et al., 2024a). have emerged as promising candidates, their compounds like tannic acid, gallic acid, eugenol, 1,3,6-Trigalloylglucose (Ou et al., 2024b), and chebulagic acid, which have been scientifically proven to effectively inhibit the growth and proliferation of this gastric pathogen. Recently, our investigation into empirical formulas and traditional formulas has uncovered several formulations that exhibit remarkable anti-HP activity, including hezi qingyou (Feng et al., 2024) and helile formula.

The helile formula (DZ), originating from the “Tai ping sheng hui fang (Taiping Holy Prescriptions for Universal Relief)”, comprises a 1:2 ratio of two primary herbs: 5 g *Sanguisorba officinalis* L., referred to as “diyu” in pinyin, and 10 g *Terminalia chebula* Retz., known as “hezi” in pinyin. According to the “Tai ping sheng hui fang (Taiping Holy Prescriptions for Universal Relief)”, DZ has been used to regulate gastrointestinal diseases and treat mild, moderate, and severe cases of diarrhea (Han, 2010). In the clinical practice of Qingyuan Traditional Chinese Medicine Hospital, it has been used as an in-hospital agreed formula for the treatment of HP infection. *Sanguisorba officinalis* L. stands out as a versatile herb utilized for the treatment of burns, hematemesis, melena, intestinal infections, and dermatitis (Jang et al., 2018). Beyond its therapeutic applications, it exhibits notable immunomodulatory properties, alongside potent antioxidant and antimicrobial effects, such as anti-HP activity (Lee et al., 2010; Shen et al., 2021). *Terminalia chebula* Retz., known as Haritaki, is a herb of profound value in Tibetan medicine, celebrated as the “king of Tibetan medicine” for its capacity toward its broad-spectrum health benefits (Ratha and Joshi, 2013). In the practice of Tibetan traditional medicine, *Terminalia chebula* Retz. is extensively utilized to combat disorders affecting the digestive, respiratory, and neurological systems (Nigam et al., 2020). Additionally, its significant anti-HP effects have been widely studied (Du et al., 2001; Malekzadeh et al., 2001; Ou et al., 2024a).

The gut microbiota plays a crucial role in regulating the immune system and maintaining gut health, and metabolomics can provide insights into the changes in metabolites within the human body. Integrating the study of gut microbiota and metabolomics can offer a more in-depth understanding of the mechanisms underlying the fight against HP infection, providing a scientific basis for drug development. Although the helile formula shows promise in the clinical treatment of gastrointestinal ailments, its *in vitro* and *in vivo* efficacy against HP infections remains undetermined, and its underlying mechanisms of action are yet to be fully elucidated.

In this study, we aimed to analyze the main components of the helile formula and explore the role of preventing and treating HP infection *in vitro* and *in vivo*. Moreover, we conducted a multi-omics analysis to further investigate the mechanism of action and identified the bacterial community and metabolites that play a crucial role in the anti-HP infection effect.

## Materials and methods

### Helile formula preparation

*Terminalia chebula* Retz (Yunnan, China, Guangzhou Zhining Pharmaceutical Co., Ltd., Lot No. 210901) and *Sanguisorba officinalis* L. (Jiangsu, China, Lot No.201902) were authenticated by Chief Pharmacist Weixing Zhu. The helile formula, specifically a composition of 5 g *Sanguisorba officinalis* L. and 10 g *Terminalia chebula* Retz, underwent a rigorous extraction process using 10-fold distilled water. It was subjected to double boiling sessions of 1.5 hours at 90°C before undergoing concentration by spin and freeze-drying. Ultimately, the formula was preserved at -20°C. The final product, helile formula, was prepared and deposited in the International Pharmaceutical Engineering Laboratory of Shandong Province.

### Component identification *via* ultra-high-performance liquid chromatography–MS/MS

Helile formula underwent a thorough analysis using UPLC-MS/MS. A YMC Triart C18 analytical column (2.1 × 100 mm, particle size 1.9 µm) was employed. The mobile phase consisted of a binary mixture of A: 0.1% formic acid in water and B: acetonitrile, implemented through a gradient profile as follows: at 0 minutes, the composition was 95% A and 5% B; at 20 minutes, it transitioned to 70% A and 30% B; remained at this ratio from 27 minutes to 27.10 minutes; and then returned to 95% A and 5% B at 40 minutes. The detection was performed at a wavelength of 270 nm.

### Chemical characterization *via* high-performance liquid chromatography

Compounds (gallic acid, ellagic acid, chebulagic acid, chebulic acid) and DZ, were analyzed using HPLC on a Waters Arc system. The system utilized an Accalim C18 column (4.6 mm x 250 mm, 5 µm) and a mobile phase consisting of acetonitrile-water (A) with 0.1% trifluoroacetic acid (B). The mobile phase gradient: 0-39 min (97% A, 3% B); 40 min (70% A, 30% B); and 41-50 min (97% A, 3% B). The mobile phase was filtered and degassed using a 0.22 µm membrane filter before usage. The flow rate was 1 mL/min, the column temperature was 25°C, and 20 µL injections were made for each analysis. Detection was performed at 270 nm.

### HP culture and infection and cell culture

RPMI 1640 medium supplemented with 10% fetal bovine serum (FBS) was utilized for the optimal growth and maintenance of GES1 cells in culture. The standard HP strains employed in our study were sourced from the American Type Culture Collection (ATCC) and clinical HP strains were kindly gifted. This sourcing information has been documented in prior research (Shen et al., 2021). To ensure their authenticity, all strains underwent authentication using morphological observation, gram staining, and biochemical reaction tests. The strains were stored at -80°C in a solution containing 65% BHI, 25% glycerol, and 10% FBS (v/v/v). For culture on Columbia agar base, the strains were grown on Columbia agar base supplemented with 5% sterile defibrinated sheep blood and incubated at 37°C in a tri-gas incubator with a gas composition of 10% CO_2_, 5% O_2_, and 85% N_2_ for three days. For liquid incubation, strains were cultured in BHI broth supplemented with 10% FBS and shaken at 150 rpm under the same gas composition described above. HP strains were cultured and stored at the School of Pharmaceutical Sciences (Shenzhen), Sun Yat-sen University.

### Detection of anti-HP activity *via* minimum inhibitory concentration assay

The MIC assay was employed by agar dilution including Mueller-Hinton agar and sheep blood (5% v/v), following the Clinical and Laboratory Standards Institute document. The procedure involves preparing agar dilution plates containing drugs such as clarithromycin or helile formula separately. An appropriate volume of bacterial culture, derived from HP that has been incubated for three days, is diluted to achieve a McFarland standard of 2.0. This diluted bacterial culture is then evenly distributed across the agar plates, with 1 to 3 microliters applied at each point. The inoculated plates are subsequently incubated in an incubator at 37°C under microaerophilic conditions characterized by 10% CO_2_, 5% O_2_, and 85% N_2_. After a 72-hour incubation period, the MIC is determined by observing the bacterial colonies grown on the plates.

### Observation of HP morphology *via* scanning electron microscopy

HP morphology observed by SEM was performed as outlined below. The ATCC 700392 strain was incubated with the drugs for 24 hours to assess morphological changes. HP was harvested by centrifugation at 6000 rpm for 3 minutes and rinsed twice with phosphate-buffered saline (PBS) for cleaning. Subsequently, the bacteria were fixed with 2.5% glutaraldehyde for an overnight period at 4°C. An ethanol dehydration sequence was employed, starting from a graded series, followed by lyophilization and final fixation. Prior to SEM examination using a Sigma 500 Scanning Electron Microscope (ZEISS, Germany), the specimens underwent metal coating.

### Inhibiting kinetics curves

The inhibition kinetics curves were constructed using the HP ATCC 700392 strain, which was subjected to varying drug concentrations. A total of 100 µl of bacterial samples were exposed to different drug doses in BHI broth supplemented with 10% FBS and then incubated in a tri-gas incubator with shaking at 150 rpm. At designated time points (0, 12, 24, 48, 60, and 72 hours), 100 µl of each sample was extracted for absorbance measurement at 600 nm.

### Analysis of HP outer membranes via N-phenyl-1-napthylamine uptake assay

To evaluate the permeabilizing potential of DZ on HP outer membranes, NPN uptake assays were conducted. The assays were carried out as follows: HP was harvested via centrifugation (6000 rpm, 5 minutes) and washed twice with 5 mM HEPES buffer (pH 7.4). The cells were then resuspended in 5 mM HEPES buffer to achieve an OD of 1 at 600 nm. Subsequently, 100 µl of the cell suspension was added to each well of 96-well microtiter plates containing 50 µl of DZ at varying concentrations. Polymyxin B served as a positive control. After adding 50 μl NPN (40 µM), fluorescence measurements were taken following a 30-minute incubation period at 37°C. The excitation wavelength was set to 350 nm, and the emission wavelength was set to 420 nm to detect the fluorescence using Cytation™ 3 (Biotek, USA).

### Detection of gene expression *via* real-time quantitative reverse transcription PCR

Total RNA extraction was methodically executed employing the RNeasy Mini Kit from Qiagen, Germany, following the manufacturer’s guidelines. The subsequent conversion of RNA to complementary DNA (cDNA) was achieved using the PrimeScript RT Master Mix from Takara, Japan. For the qRT-PCR analysis, a precisely controlled amount of cDNA (100 ng) was employed, along with TB Green^®^ reagent (Takara, Japan), on the state-of-the-art Applied Biosystems 7500 Real-Time PCR System from Thermo Fisher Scientific, USA. The primer sequences utilized in this qRT-PCR assay were sourced from BBI Life Sciences and are detailed in **Table 1**.

**Table 1:** The primers of RT-QPCR.

Gene name	Sequences forward(F)	Sequences reverse(R)
16S rRNA	CCGCCTACGCGCTCTTTAC	CTAACGAATAAGCACCGGCTAAC
flaA	ATTGGCGTGTTAGCAGAAGTGA	TGACTGGACCGCCACATC
flaB	ACATCATTGTGAGCGGTGTGA	GCCCCTAACCGCTCTCAAAT
babA	TGCTCAGGGCAAGGGAATAA	ATCGTGGTGGTTACGCTTTTG
alpA	GCACGATCGGTAGCCAGACT	ACACATTCCCCGCATTCAAG
alpB	ACGCTAAGAAACAGCCCTCAAC	TCATGCGTAACCCCACATCA
ureE	TCTTGGCTTGGATGTGAATG	GGAATGGTTTGAAACGAGGA
ureF	GGGGCTTGTGGATAGCATAA	CGCATTCTTTTGGGCTAGAA

### Functional prediction *via* network pharmacology

The potential target genes for hezi and diyu were sourced from The Encyclopedia of Traditional Chinese Medicine (ETCM, accessible at http://www.tcmip.cn/ETCM). The HP target genes were retrieved from Genecards (www.genecards.org). A Venn Diagram was generated online using the Bioinformatics Resource Portal at Ghent University (https://bioinformatics.psb.ugent.be/webtools/Venn/) to visualize the gene overlap. The protein-protein interaction (PPI) network analysis was conducted with the STRING database in its 2023 update (https://cn.string-db.org/cgi/input?sessionId=b4apxqd43I6Z&input_page_show_search=on). The Reactome Pathway analysis used the Analysis Tools platform (https://reactome.org/).

### Cell viability and cell adhesion activity

Cell Counting Kit-8 (CCK-8) assay (Beyotime, Shanghai, China) was conducted to detect the cell viability. 10,000 GES-1 cells were seeded and incubated overnight for attachment and growth. The cells were then exposed to the drug for 24 hours. Post-incubation, CCK-8 solutions were added to the wells and incubated for 2 hours. A microplate reader (Multiskan GO, Thermo Scientific, USA) was used to detect the optical density at 450 nm. To assess cell adhesion activity, HP was introduced to GES-1 cells at a multiplicity of infection (MOI) of 100:1, in the presence of varying concentrations of DZ (0, 40, 80 µg/mL) for 6 hours. Then the GES-1 cells were rinsed twice with PBS. Urea test solutions were then introduced to evaluate the intracellular urea levels of HP, and the optical density at 560 nm (OD560) was measured using a microplate reader (Multiskan GO, Thermo Scientific, USA). For the observation of cell adhesion, GES-1 cells were co-cultured with FITC-labeled HP at a ratio of 1:100, along with 80 µg/mL DZ for 6 hours. The cells were then washed twice with PBS, and the FITC intensity was detected using confocal microscopy (Olympus, Japan).

### Nitric oxide activity

After incubating together for 6 hours, the cell culture supernatant was gathered to measure NO activity using the Griess Reagent System kit, following the manufacturer’s instructions. Griess reagents (sulfanilamide and N-(1-naphthyl)ethylenediamine dihydrochloride) were then added. Finally, the nitrite levels in the samples were quantified based on the standard curve by measuring the optical density at 540 nm using a microplate reader (Multiskan GO, Thermo Scientific, Waltham, MA, USA).

### Procedure of animal experiments

The animal experiments were performed in the ABSL-2 Laboratory of the Experimental Animal Center at the Shenzhen Campus of Sun Yat-sen University, with ethical approval granted under number 2023003411 and an animal use license issued with the number SYSK (Guangdong) 2021-0112 and adhered to the Reporting *In Vivo* Experiments (ARRIVE) guidelines. Mice were kept in a controlled environment devoid of specific pathogens, with a temperature maintained at 25 ± 1°C, and were allowed unrestricted access to food and water. Following an adaptive feeding period, a total of 24 specific pathogen-free (SPF) male mice (KM male mice, 16-18 g, origin: Sun Yat-sen University (Experimental Animal Center, East Campus) (SCXK (Yue) 2021-0029)) were randomized into two groups: a control group (n = 6) and a model group (n = 18). The control mice were nourished with a diet containing 10% FBS (fetal bovine serum), whereas the model group underwent five feeding sessions with HP. After two weeks of HP infection and colonization, the model group was further subdivided into three distinct groups: HP (n = 6), Positive (n = 6), and DZ (n = 6). The HP group received water, the Positive group was administered a cocktail of three antibiotics (Amoxicillin, Omeprazole, and CLA). According to the clinical daily dose, the dosage for mice was calculated using the body surface area method, the DZ group was given 428 mg/kg of DZ for 10 days. Subsequently, following the final drug administration, fresh fecal samples were carefully collected from each mouse and stored at −80°C to preserve for analysis. Ultimately, the mice were euthanized, and various tissues were harvested for further study.

### Detection of anti-HP activity *via* urea fast test

Gastric tissues from mice were first rinsed with PBS to remove residual debris. Following the washing step, the tissues were then cut into smaller pieces and submerged in a urea solution. The urea-treated tissue samples were subsequently placed in a tri-gas incubator for 12 hours. After the incubation period, the tissues were photographed for further analysis.

### Hematoxylin and eosin staining for mice gastric mucosa examination

Mouse gastric tissues were first washed with PBS to remove any residual material. Following this, the tissues were fixed in 4% paraformaldehyde for 24 hours to preserve their structure. After fixation, the samples underwent dehydration processes using a series of graded ethanol solutions (75%, 85%, 95%, and 100%). Once dehydrated, the tissues were cleared using dimethylbenzene and then embedded in paraffin wax for subsequent hematoxylin and eosin (H&E) staining.

### Quantitative analysis of inflammatory factors via enzyme-linked immunosorbent assay

Mouse gastric tissues were initially rinsed with PBS to cleanse them of any contaminants. Subsequently, the tissues were processed by grinding and then subjected to centrifugation to isolate the supernatant. The collected supernatant was then analyzed for specific analyte levels using an ELISA kit, in accordance with the manufacturer’s guidelines. The absorbance of the samples was measured at a wavelength of 450 nm using Cytation™ 3 microplate reader (Biotek, USA).

### Detection of protein expression *via* Western blot

The protein extraction is performed using RIPA Buffer (Beyotime, China), followed by protein concentration determination using the BCA Protein Assay Kit (Beyotime, China). Subsequently, 30 μg of the extracted proteins are separated using SDS-PAGE gels and transferred onto a membrane. The membrane is then blocked with skimmed milk and incubated with primary antibodies overnight. The following day, the membrane is incubated with corresponding secondary antibodies. Finally, the protein bands are visualized using Beyo ECL (Beyotime, China) in a Chemi Scope 6200 Visualizer, from Clinx Science Instruments.

### 16S ribosomal DNA sequencing analysis

Mouse fecal samples underwent 16S rDNA sequencing utilizing the PE250 method on either the Illumina or MGI platforms provided by Guangdong Magigene Biotechnology Co., Ltd. in Guangzhou, China. The sequencing was conducted with specific 16S primers, namely 515F, and 806R, to amplify and analyze the bacterial DNA present in the samples.

### Metabolomics analysis

Mouse fecal samples were gradually thawed at 4°C, and an appropriate sample (50-100 mg) was precisely weighed into a centrifuge tube. 0.2 mL of pre-chilled water was added and homogenized for 60 seconds. Subsequently, 0.8 mL of a pre-chilled extraction solution (a 1:1 v/v mixture of methanol and acetonitrile) was added and again homogenized for 60 seconds. The mixture was then subjected to low-temperature ultrasonic extraction for 30 minutes. Afterward, it was allowed to stand for 1 hour at -20°C to facilitate protein precipitation. The sample was centrifuged at 12,000 rpm for 10 minutes at 4°C, and the supernatant was dried under vacuum. The residue was reconstituted with 0.2 mL of a 30% acetonitrile solution, homogenized, and centrifuged at 14,000 rpm for 15 minutes at 4°C. The resulting supernatant was collected and analyzed using a UPLC-Orbitrap-MS system (UPLC, Vanquish; MS, HFX). The analytical conditions for UPLC included a Waters HSS T3 column (100×2.1 mm, 1.8 μm) maintained at 40°C, with a flow rate of 0.3 mL/min and an injection volume of 2 μL. The solvent system comprised phase A (Milli-Q water with 0.1% formic acid) and phase B (acetonitrile with 0.1% formic acid), following a gradient program from 0 to 1 min (100:0, v/v), 1 to 12 min (100:0 to 5:95, v/v), 12 to 13 min (5:95, v/v), 13 to 13.1 min (5:95 to 100:0, v/v), and 13.1 to 17 min (100:0, v/v). High-resolution MS data were acquired using a Q Exactive HFX Hybrid Quadrupole Orbitrap mass spectrometer equipped with a heated ESI source (Thermo Fisher Scientific), employing the full-ms-ddMS2 acquisition method. The ESI source parameters were adjusted to a sheath gas pressure of 40 arb, aux gas pressure of 10 arb, spray voltage of +3000 V/-2800 V, temperature of 350°C, and ion transport tube temperature of 320°C. The primary mass spectrometry scanning range was set from 70 to 1050 Da, with a primary resolution of 70,000 and a secondary resolution of 17,500. Raw MS data were collected using Xcalibur 4.1 (Thermo Scientific) and processed with Progenesis QI (Waters Corporation, Milford, USA). Quantified data were formatted into Excel. The data were analyzed using R packages, undergoing multivariate data analysis partial least-squares discriminant analysis (PL-SDA). The variable importance in the projection (VIP) value was calculated for each variable in the PLSDA model to assess its contribution to classification. Metabolites with a VIP value >1 were further analyzed using Student’s t-test at the univariate level, with p values <0.05 considered statistically significant.

### Statistical analysis

The data were visualized using GraphPad Prism 8 software. Statistical analyses were conducted using the two-tailed Student’s t-test or one-way ANOVA, followed by appropriate *post-hoc* analyses. Data displayed as the mean +/− an estimate of variability (SD, SEM) of three independent experimental replications. The level of statistical significance was represented by asterisks, with * indicating *P* < 0.05 and ** indicating *P* < 0.01, respectively.

## Results

### The chemical constituents of helile formula were analyzed using UPLC-MS/MS and the consistency of the constituent levels was evaluated *via* HPLC techniques

**Figure 1:**
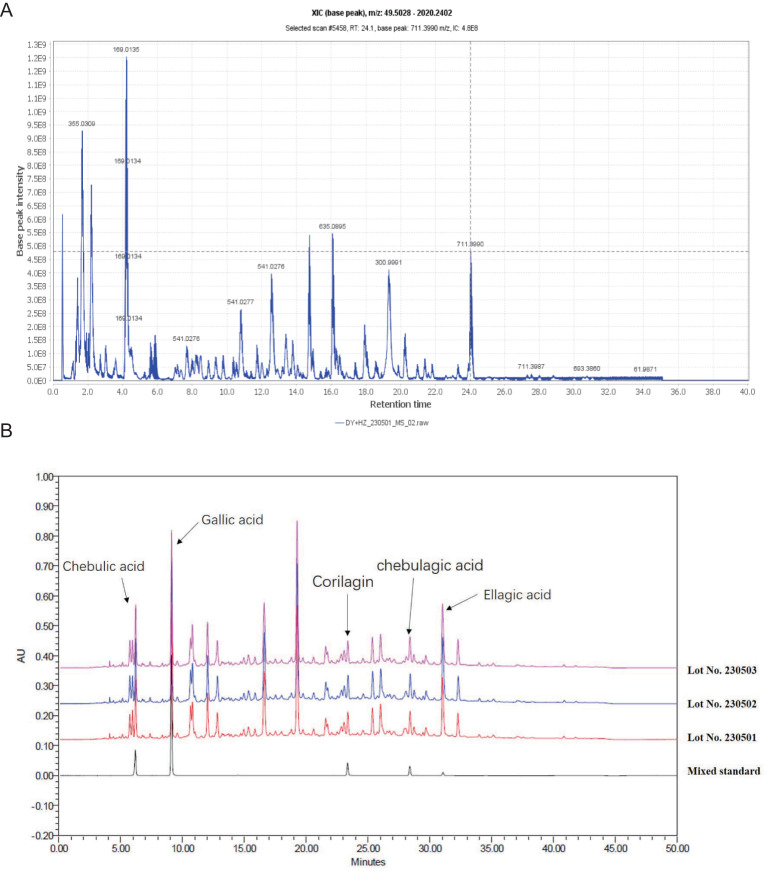
The chemical constituents of helile formula (DZ) were analyzed using a combination of Ultra-Performance Liquid Chromatography-Tandem Mass Spectrometry (UPLC-MS/MS) and High-Performance Liquid Chromatography (HPLC) techniques. **(A)** The base peak XIC chromatogram of DZ was analyzed by UPLC-MS/MS. **(B)** The HPLC profiles of these three batches of DZ.

The initial step involved the identification of various compounds within the extracts by employing UPLC-MS/MS, which facilitated the determination of precise mass characteristics, molecular formula, and MS/MS fragmentation patterns, all supported by relevant literature. The base peak chromatogram under negative ionization conditions is depicted in **Figure 1A** and **Table 2**. To establish the consistency of the constituent levels, three distinct batches of DZ were independently prepared and subjected to HPLC analysis. The HPLC profiles of these three batches are presented in **Figure 1B**, with the detailed quantitative findings summarized in **Table 3**.

**Table 2:** The information of HR-LCMS of helile formula (DZ).

NO.	RT (min)	[M-H]-	Formula	Name
1	1.40	191.0553	C_7_H_12_O_6_	Quinic acid
2	1.64	355.0309	C_14_H_12_O_11_	Chebulic acid
3	3.70	783.0685	C_34_H_24_O_22_	Terflavin B
4	4.20	169.0134	C_7_H_6_O_5_	Gallic acid
5	7.60	1083.0592	C_48_H_28_O_30_	Terchebulin
6	9.70	483.0786	C_20_H_20_O_14_	3,6-Digalloylglucose
7	10.75	1083.0587	C_48_H_28_O_30_	Punicalagin α
8	12.54	1083.0587	C_48_H_28_O_30_	Punicalagin β
9	14.80	633.0751	C_27_H_22_O_18_	Corilagin
10	14.90	651.0862	C_27_H_24_O_19_	Chebulanin
11	16.10	635.0895	C_27_H_24_O_18_	1,3,6-Trigalloylglucose
12	18.00	953.0890	C_41_H_30_O_27_	Chebulagic acid
13	19.30	300.9991	C_14_H_6_O_8_	Ellagic acid
14	20.20	955.1083	C_41_H_32_O_27_	Chebulinic acid

**Table 3:** Calibration curves and contents of the compounds of DZ.

Compounds	Regression equation	R^2^	Linear range (μg/mL)	Content (100%)
Ellagic acid	y = 33,222,605.97 x - 100,548.64	1.00	14.18-1418.40	2.14 ± 0.07
Gallic acid	y = 63,216,824.77 x + 20,924.70	1.00	13.18-65.89	1.69 ± 0.01
Chebulagic acid	y = 27,250,898.87 x + 3,284.88	1.00	3.02-30.23	0.86 ± 0.05
Chebulic acid	y = 16,884,099.74 x - 1,754.24	1.00	12.47-74.81	2.78 ± 0.02
Corilagin	y = 36,131,009.44 x + 8,504.40	1.00	2.75-13.77	0.55 ± 0.01

### *In vitro* antibacterial activities of helile formula and its effects on HP growth

**Figure 2:**
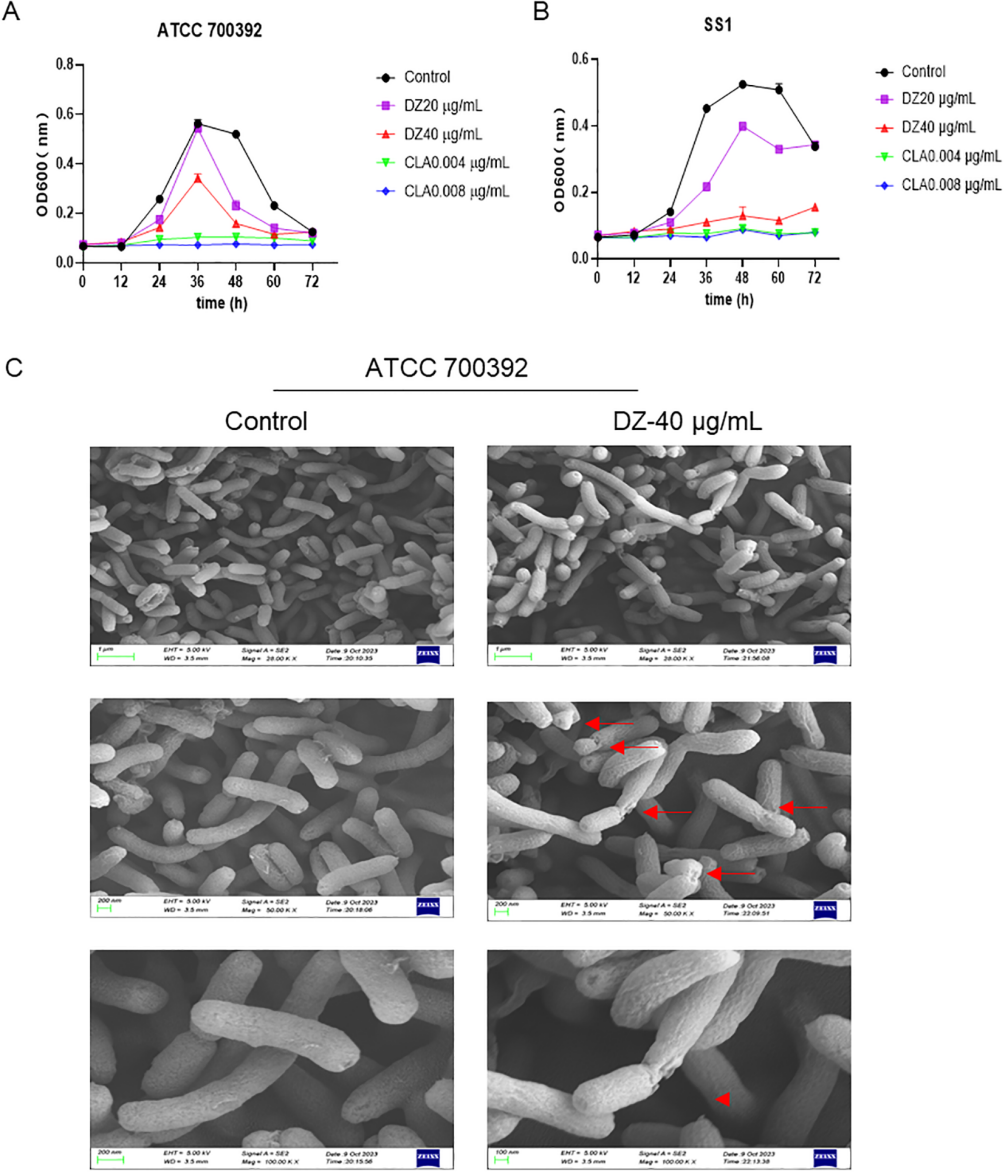
*In vitro* antibacterial activities of helile formula (DZ) and its effects on *Helicobacter pylori* (HP) growth. **(A, B)** The kinetics of HP growth inhibition for the ATCC 700392 and SS1 strains following treatment with varying concentrations of DZ (0, 20, 40 µg/ml) and positive drug clarithromycin(CLA, 0.004, 0.008 µg/ml). **(C)** The morphological changes in the HP ATCC 700392 strain after exposure to a concentration of 40 µg/ml of DZ by scanning electron microscopy (SEM).

The antibacterial efficacy of helile formula against multiple strains of HP (standard strains and clinical isolates) was assessed by determining MICs using agar dilution (**Table 4**). The MICs of helile formula against various HP strains ranged from 40 to 320 μg/ml. Growth inhibition kinetics curves demonstrated that 40 μg/ml of helile formula effectively suppressed HP growth (**Figure 2A, B**). Scanning electron microscopy (SEM) images illustrated that the cell surfaces of bacteria in the control group exhibited smooth, uniform, and curved rod-like shapes, while those treated with helile formula displayed damaged and shriveled surfaces (**Figure 2C**).

**Table 4:** MIC values for multiple HP strains of DZ.

Type	HP strains	MIC of DZ (μg/mL)	MIC of clarithromycin (μg/mL)
Standard	ATCC700392	40	0.008
Standard	SS1	40	0.008
Standard	ATCC43504	40	0.008
Clinical	CS01	320	> 0.064
Clinical	ICDC11101	40	> 0.064
Clinical	QYZ001	40	0.064
Clinical	QYZ003	40	> 0.064
Clinical	QYZ004	80	> 0.064

Clarithromycin serves as the positive control.

### The impact of helile formula on bacterial outer membrane permeability, colonization and motility, and cell adhesion

**Figure 3:**
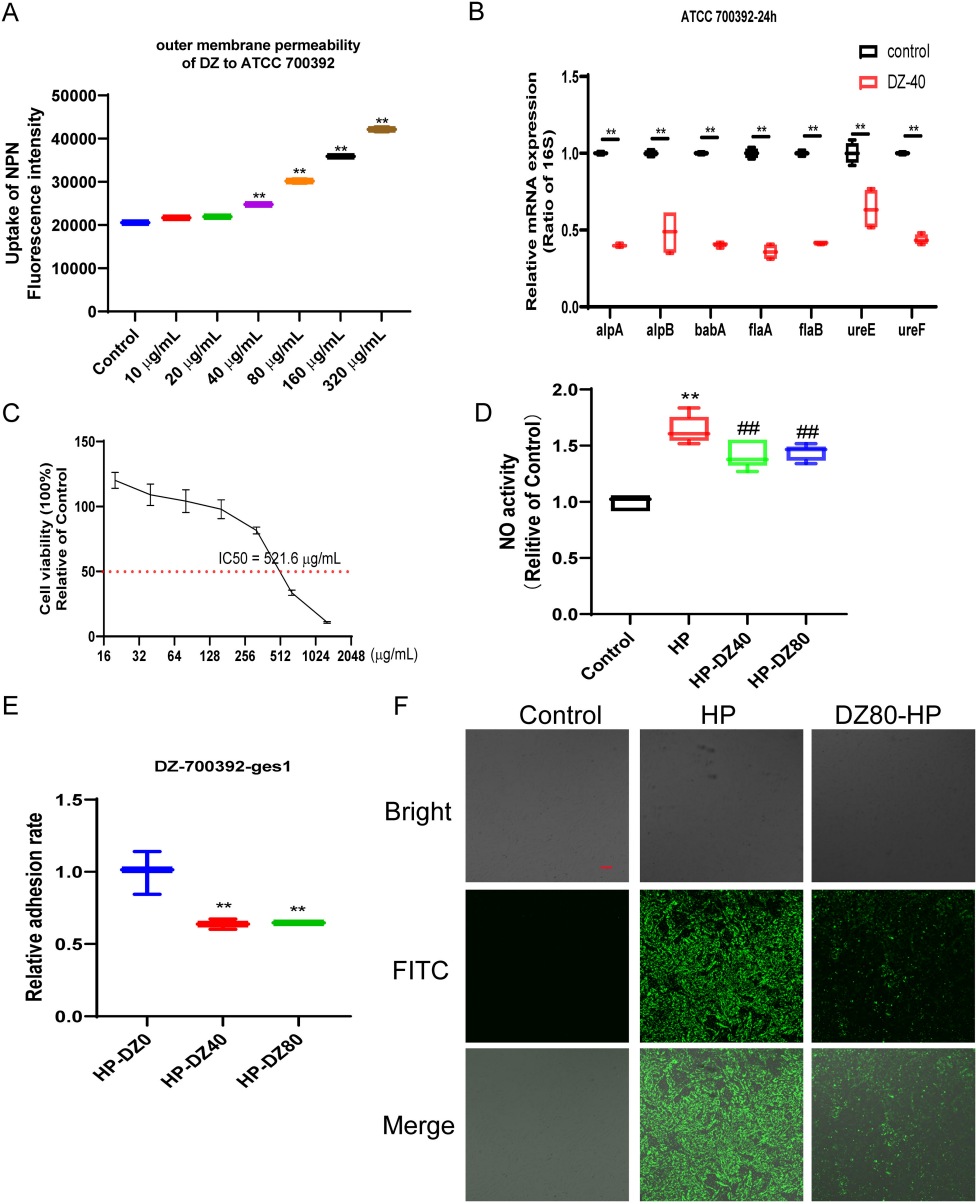
The Impact of helile formula (DZ) on bacterial outer membrane permeability, colonization and motility, and cell Adhesion *in vitro.* **(A)** ATCC 700392 outer membrane permeability was assessed. **(B)** The mRNA expressions of alpA, alpB, babA, flaA, flaB, ureE, and ureF of ATCC 700392 were detected. **(C)** The IC50 of DZ on GES1 cells was measured. **(D)** The Nitric Oxide (NO) Activity was measured. **(E)** The adhesion rate of ATCC 700392 on GES1 cells was measured by urea test solution. **(F)** The adhesion rate of ATCC 700392 on GES1 cells was observed by a Confocal Laser Scanning Microscope (100X). Scale bar = 1 µm. N-phenyl-1-napthylamine, NPN; helicobacter pylori, HP. The level of statistical significance was represented by asterisks, with * or # indicating P < 0.05 and ** or ## indicating P < 0.01, respectively.

As depicted in **Figure 3A**, the uptake of NPN was notably enhanced by helile formula concentrations ranging from 40 to 320 μg/ml. Helile formula demonstrated a pronounced enhancement in the permeability of bacterial outer membranes. This enhancement is likely attributable to the disruption of the membrane’s structural integrity, thereby impairing its functional capabilities. Furthermore, 40 μg/ml helile formula exhibited a substantial inhibitory effect on the expression of adhesion-related genes, such as alpA, alpB, and babA, concurrently leading to a decrease in the expression of urease genes like ureE and ureF, as well as the flagellins encoded by flaA and flaB (**Figure 3B**). This suggests a potential mechanism by which helile formula may impair bacterial colonization and motility. Furthermore, the IC50 value of helile formula on GES1 was determined to be 521.6 μg/ml, as depicted in **Figure 3C**. To delve deeper into its mechanisms of action, we conducted co-culture experiments with bacterial cells, which revealed that helile formula effectively curbed the activation of NO by HP and significantly reduced bacterial adhesion (**Figures 3D, E**). To provide a more tangible observation, we labeled HP with FITC before infection, and subsequent analysis showed that helile formula markedly inhibited the attachment of FITC-labeled HP to host cells (**Figure 3F**). In summary, these findings suggest that the antibacterial activity of helile formula may be mediated by modulating the permeability of the bacterial outer membrane, influencing adhesion genes, urease genes, flagellins and exerting an anti-adhesive effect.

### Network pharmacology analysis of helile formula’s antibacterial role

**Figure 4:**
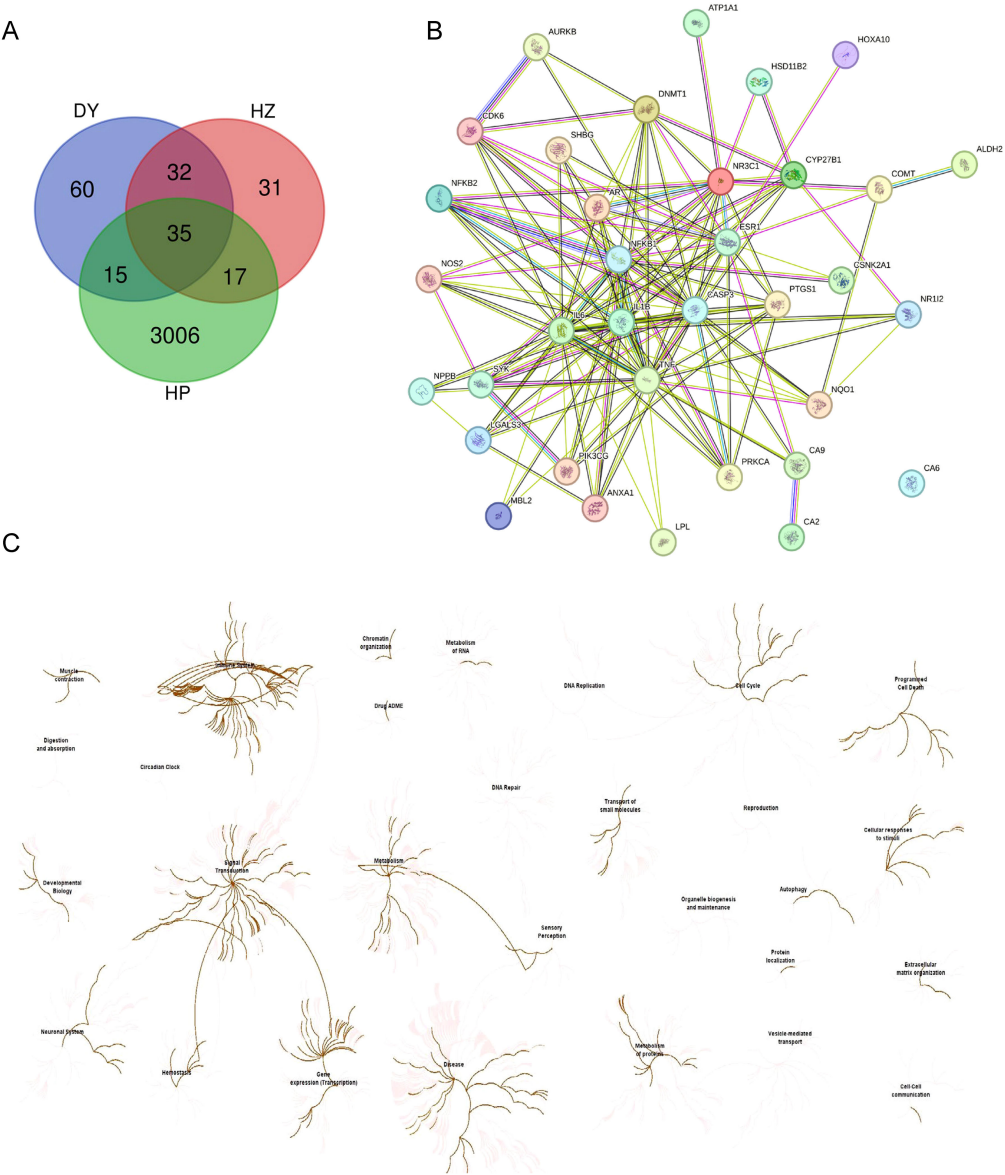
Network pharmacology analysis of the antibacterial role of helile formula (DZ) and validate its targets associated with regulated pathways. **(A)** Venn Diagram of target genes of Diyu (DY), Hezi (HZ), and *Helicobacter pylori* (HP). **(B)** The 35 co-target genes of DY, HZ, and HP were displayed by the protein-protein interaction (PPI) network. **(C)** The Reactome Pathway was analyzed by target genes from DY, HZ, and HP.

In our investigation of helile formula’s antibacterial mechanism, we initially gathered targets from diyu, hezi, and HP, and visualized their shared targets using a Venn diagram, as illustrated in **Figure 4A**. A PPI network, featuring 35 co-expressed targets, was constructed in **Figure 4B** to further substantiate these findings. By conducting a Reactome pathway analysis, we identified key pathways, including Gene Expression (Transcription), RNA Polymerase II Transcription, Generic Transcription Pathway, Cytokine Signaling in the Immune System, and Interleukin-mediated Signaling (**Figure 4C**).

### Anti-HP activity and anti-inflammation role of helile formula *in vivo*

**Figure 5:**
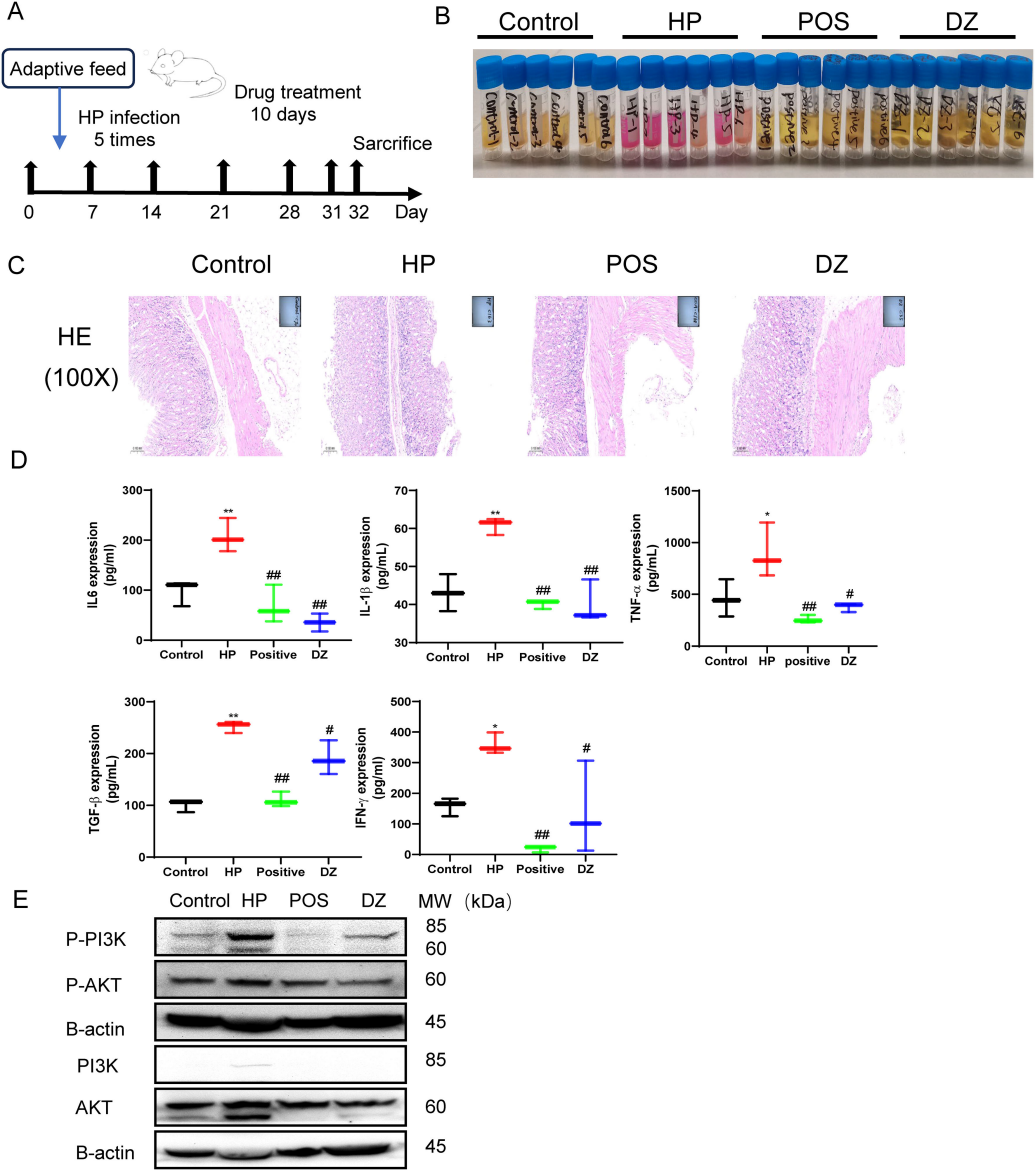
Anti-*Helicobacter pylori* (HP) activity and anti-inflammation role of helile formula (DZ) *in vivo.* **(A)** The workflow of animal experiments. **(B)** The fast urea test. These gastric tissues were collected from four groups, the control group, the *Helicobacter pylori* model group (HP), the Positive group (POSITIVE), and the helile formula treatment group (DZ). **(C)** Hematoxylin and Eosin staining (200X). **(D)** These protein expressions (IL6, IL1β, TNF-α, TGF-β and IFN-γ) were analyzed by Enzyme-linked immunosorbent assay (ELISA). **(E)** The expression of PI3K, p-PI3K, AKT and p-AKT proteins was detected by western blot. The level of statistical significance was represented by asterisks, with * or # indicating P < 0.05 and ** or ## indicating P < 0.01, respectively.

To comprehensively investigate the biological effects of DZ in antibacterial and anti-inflammatory actions, we carefully designed an *in vivo* experiment, as illustrated in **Figure 5A**. At the beginning of the experiment, the animals were divided into multiple cycles, undergoing five rounds of HP infection and colonization, followed by a ten-day treatment with helile formula. During the experiment, a rapid urease test was performed on the gastric tissues of mice, showing no pink reaction in the control group and the group receiving helile formula treatment, while the gastric mucosa of the HP-infected group exhibited inflammatory features (**Figure 5B**). Furthermore, HE staining analysis demonstrated that in the control group, the gastric mucosa presented a normal appearance with orderly-arranged cells and glands, and there was no sign of inflammation. In the HP-infected group, the gastric mucosa exhibited disordered epithelial cells, cell exfoliation, a significant infiltration of inflammatory cells, and damaged glandular structures. In the positive-treatment group, the arrangement of cells was more regular, the degree of inflammation was decreased, and the glandular structures showed signs of recovery. In the DZ-treated group, the cell arrangement still had a certain degree of disorder, the infiltration of inflammatory cells was reduced, and the glandular structures had partially recovered, indicating that the DZ treatment could alleviate the damage caused by HP infection (**Figure 5C**). ELISA testing on the gastric tissue showed significantly elevated expression of inflammatory factors in the HP-infected group, including IL-6, IL-1ß, TNF-α, TGF-β, and IFN-γ. In contrast, data from the helile formula treatment group and the positive control group indicated that the helile formula could significantly reverse the activity of inflammatory factors induced by HP infection, highlighting its potent anti-inflammatory effects (**Figure 5D**). As shown in **Figure 5E**, western blot analysis demonstrated that HP infection could induce the up-regulation of the expression levels of P-PI3K, P-AKT, PI3K, and AKT proteins in the gastric tissues of mice. Both the positive treatment group and the DZ treatment group exhibited inhibitory effects on the expression of these four proteins. Overall, the treatment with DZ was capable of suppressing the activation of the PI3K/AKT pathway induced by HP infection in the gastric tissues of mice.

### Helile formula regulated the composition of gut microbiota

**Figure 6:**
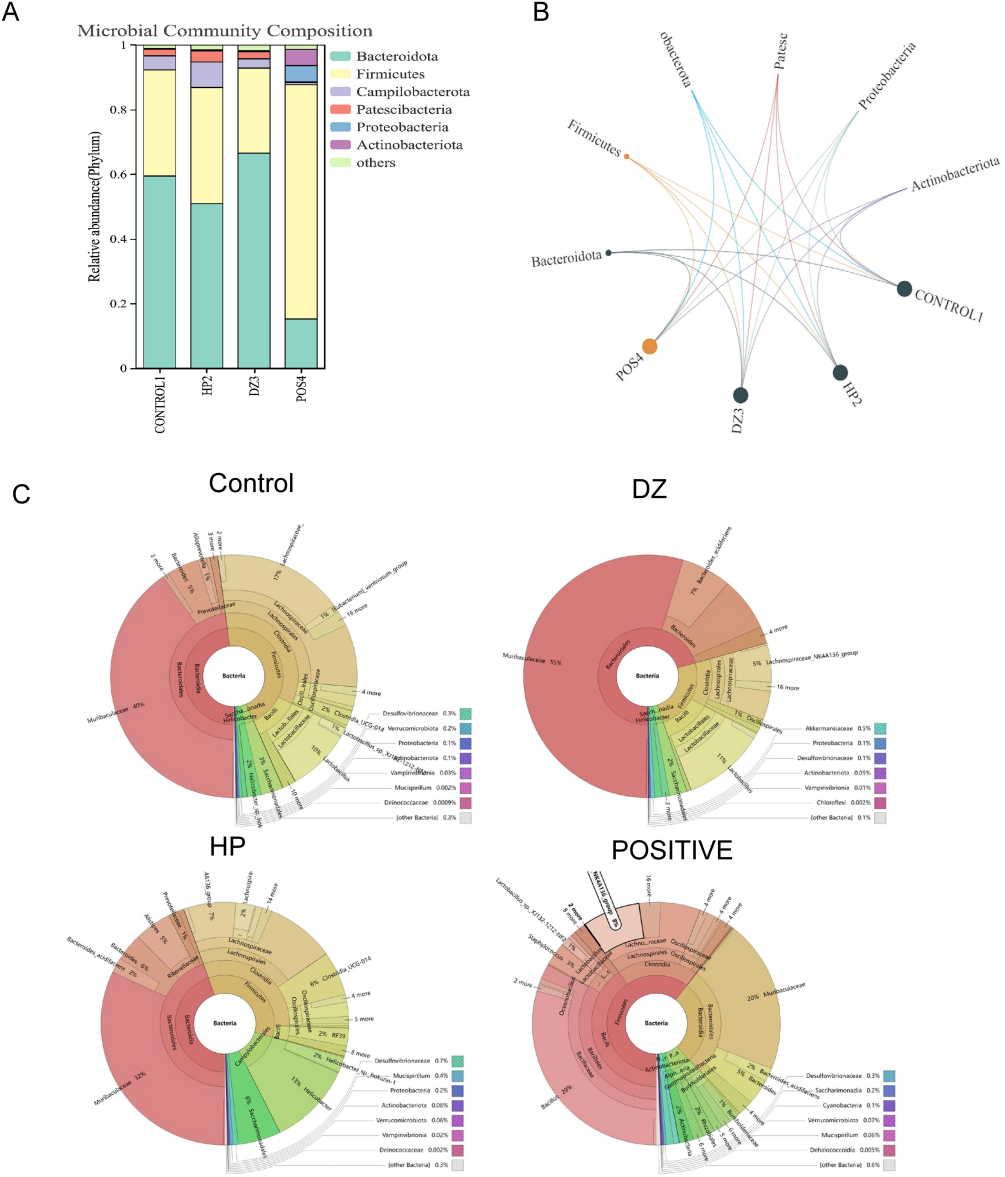
Helile formula (DZ) regulated the composition of gut microbiota. **(A, B)** microbial community composition at the phylum level. **(C)** bacterial phylogenetic tree. All were from the control group, the *Helicobacter pylori* model group (HP), the Positive group (POSITIVE), and the helile formula treatment group (DZ).

According to **Figures 6A, B**, the microbial community composition at the phylum level in the HP group exhibited changes compared to the control group, with a decrease in *Bacteroidota* and an increase in *Firmicutes*, *Campylobacterota*, and *Patescibacteria*. However, the positive group showed a decrease in *Bacteroidota* and an increase in *Firmicutes*, *Proteobacteria*, and *Actinobacteria*. These results suggest that antibiotic treatment can disrupt the normal ecological balance of the gut microbiota, leading to microbial community disruption and a failure to recover to its original state. The helile formula group was similar to the control group, indicating that helile formula may improve microbial metabolism. Upon analyzing the bacterial phylogenetic tree in **Figure 6C**, it is evident that *Muribaculaceae* populations were reduced in the HP and Positive groups, whereas the helile formula group showed an increase in *Muribaculaceae* populations. This suggests that *Muribaculaceae* may play a crucial role in the anti-inflammatory and antibacterial effects of DZ.

### Helile formula regulated HP affected-differential metabolites

**Figure 7:**
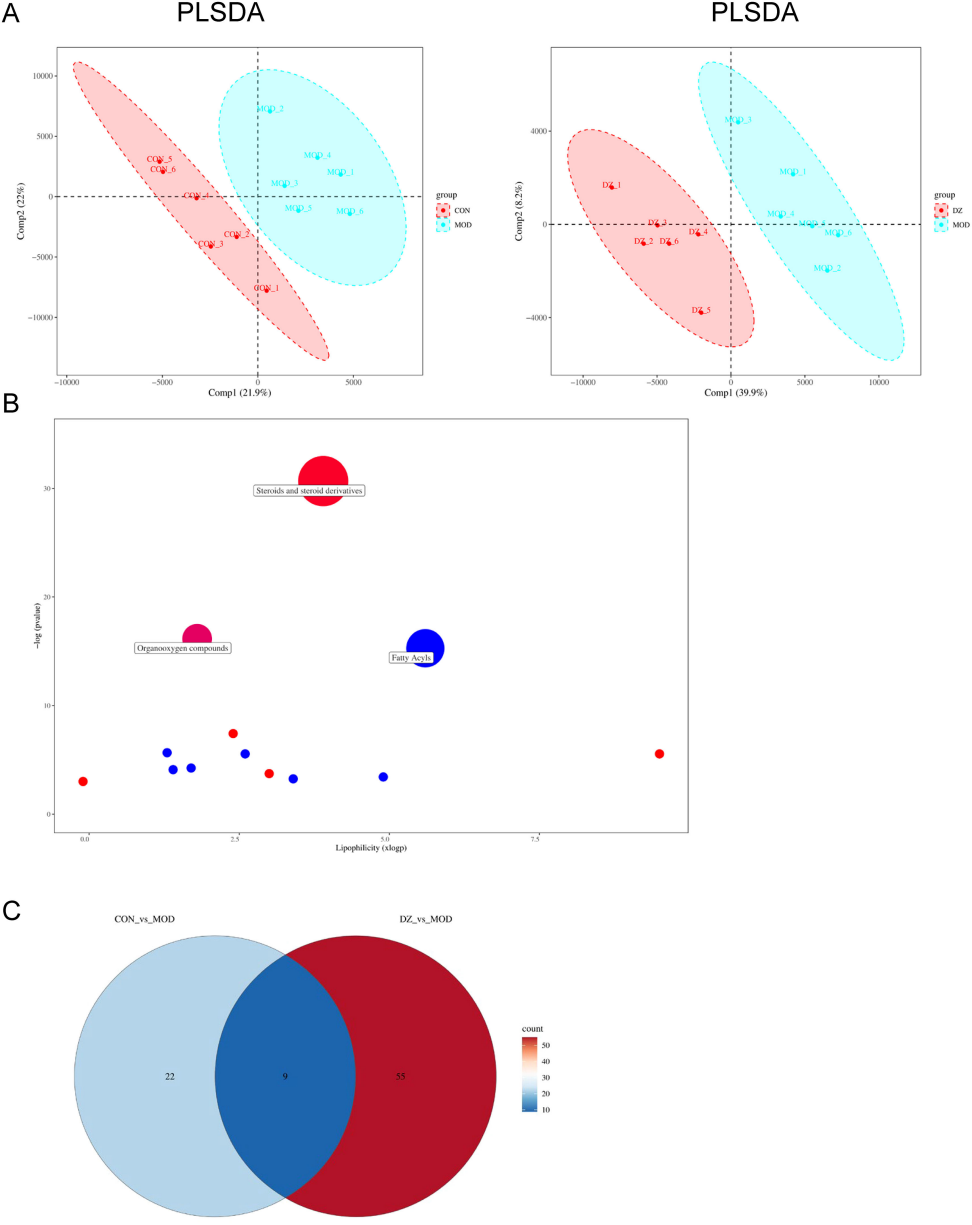
Helile formula (DZ) regulated HP affected-differential metabolites. **(A)** Partial least squares discriminant analysis (PLS-DA) plots. **(B)** Metabolic pathway plot. **(C)** Venn plot. All were from the control group, the *Helicobacter pylori* model group (HP), the Positive group (POSITIVE), and the helile formula treatment group (DZ).

**Figure 8:**
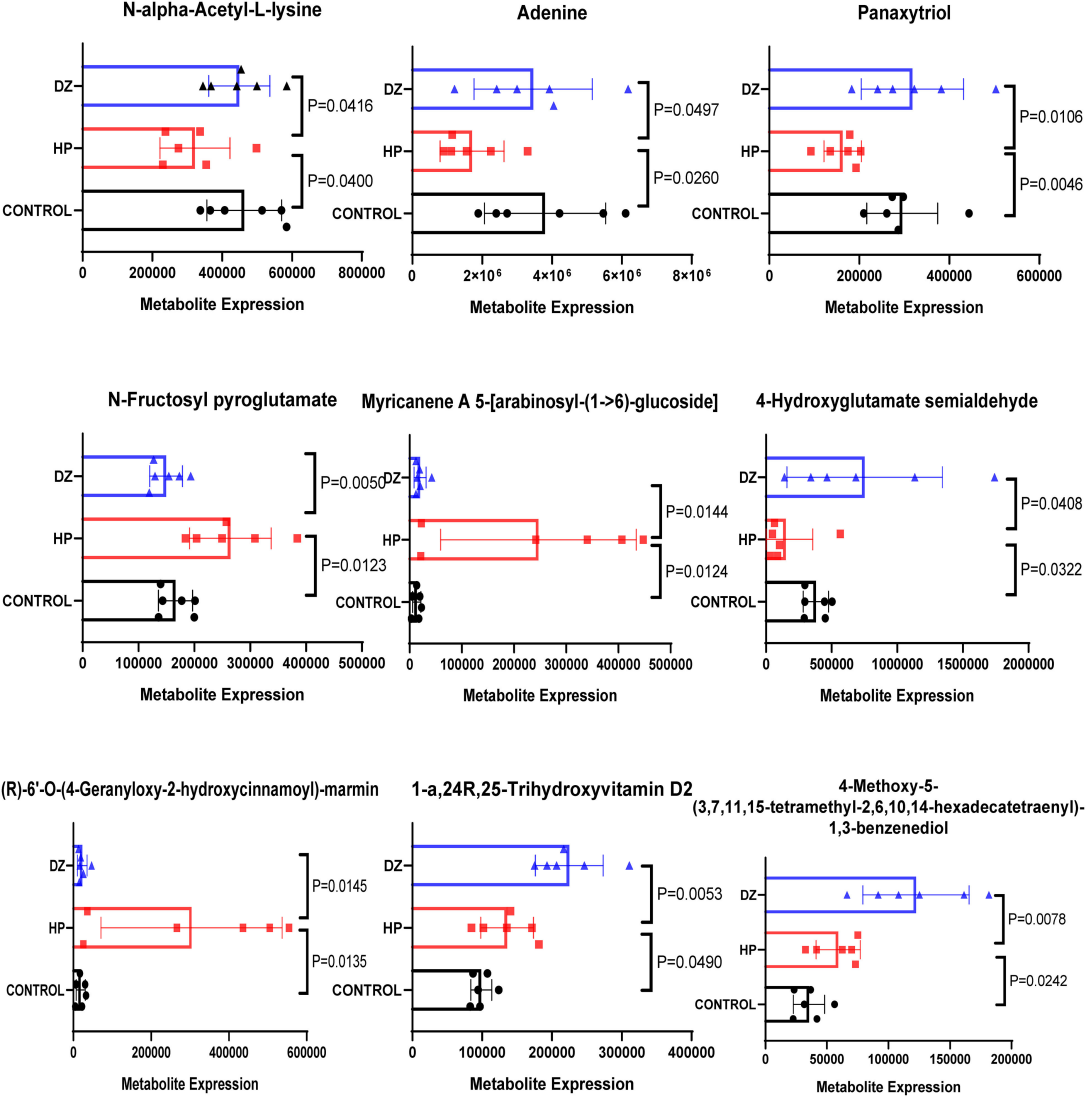
Helile formula (DZ) regulated *Helicobacter pylori* (HP)-affected differential metabolites. Nine differential metabolites that met both VIP > 1 and p < 0.05 were identified as the key metabolites associated with helile formula (DZ)-regulated *Helicobacter pylori* (HP) infection.

Variables exhibiting a coefficient of variation (CV) ≤ 30% were chosen for PLS-DA to assess the metabolic changes induced by HP infection and the modulation of helile formula (**Figure 7A**). The PLS-DA score plot demonstrated a clear separation between the control and model groups, indicating alterations in their metabolic profiles. The separation between the helile formula group and the model group suggested that helile formula intervention had a regulatory effect on the metabolic abnormalities induced by HP infection. Enrichment analysis further revealed the most significant metabolic pathway associated with HP infection and the efficacy of helile formula, including steroids and steroid derivatives, organoxygen compounds, and fatty acyls (**Figure 7B**). Nine differential metabolites that met both VIP > 1 and p < 0.05 were identified as the key metabolites associated with helile formula-regulated HP infection (**Figure 7C**), including 1-a,24R,25-Trihydroxyvitamin D2, 4-Methoxy-5-(3,7,11,15-tetramethyl-2,6,10,14-hexadecatetraenyl)-1,3-benzenediol, (R)-6’-O-(4-Geranyloxy-2-hydroxycinnamoyl)-marmin, 4-Hydroxyglutamate semialdehyde, N-Fructosyl pyroglutamate, Panaxytriol, Adenine, N-alpha-Acetyl-L-lysine and Myricanene A 5-[arabinosyl-(1->6)-glucoside]. As depicted in **Figure 8**, the HP treatment model group exhibited a reduction in N-alpha-Acetyl-L-lysine, Adenine, 4-Hydroxyglutamate semialdehyde, and Panaxytriol. In contrast, the helile formula treatment was effective in enhancing the levels of these metabolites. Conversely, HP treatment resulted in the upregulation of five other metabolites. However, helile formula treatment was only capable of reversing the effects on three specific metabolites, namely N-Fructosyl pyroglutamate, Myricanene A 5-[arabinosyl-(1->6)-glucoside], and (R)-6’-O-(4-Geranyloxy-2-hydroxycinnamoyl)-marmin. These findings indicate that helile formula can effectively modulate seven crucial metabolites to mitigate the damage caused by HP.

### Differential metabolite adenine recovered the HP-induced GES1 damage, alleviated the HP adhesion, and suppressed the HP-induced inflammation

**Figure 9:**
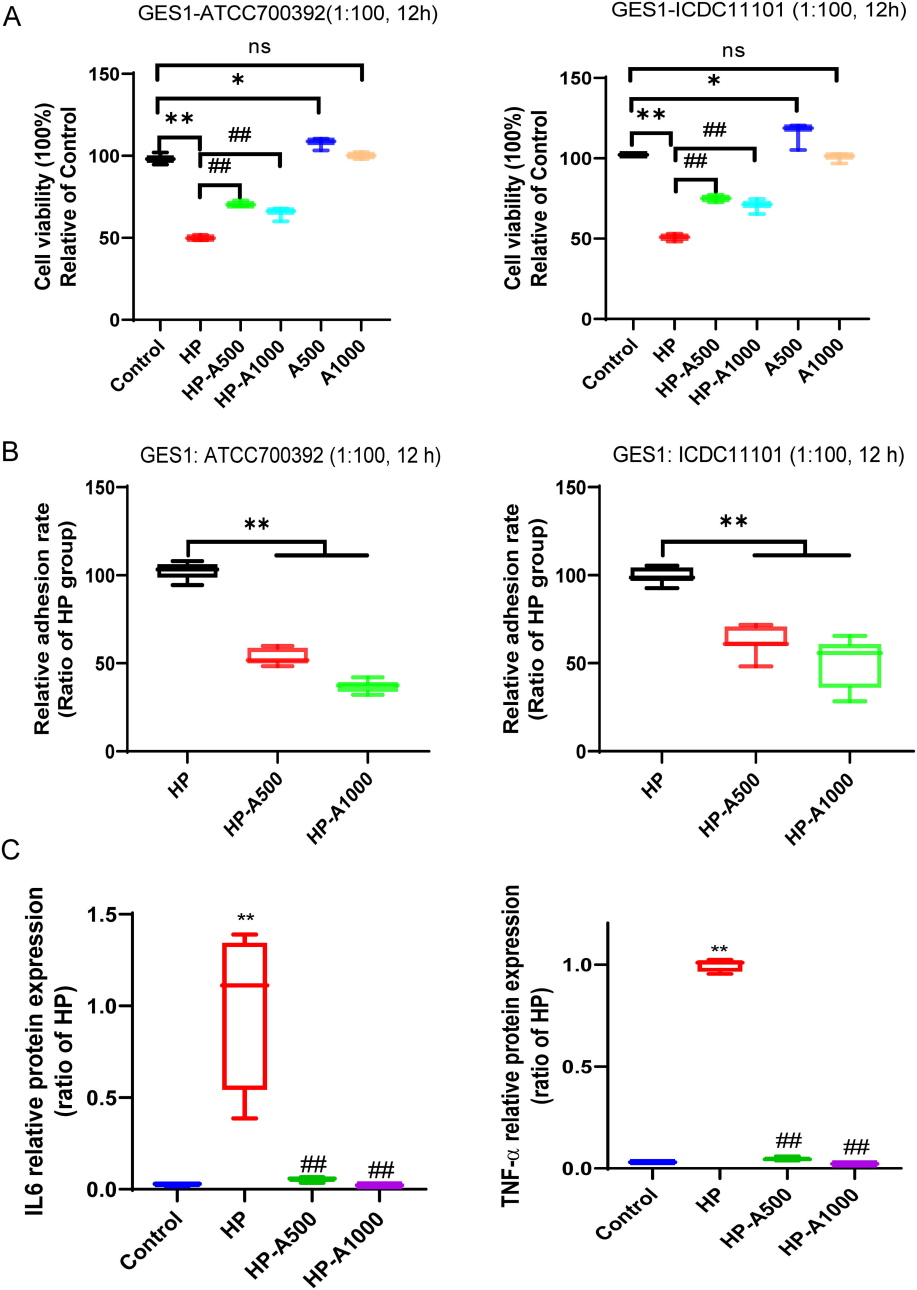
Differential metabolite adenine recovered the HP-induced GES1 damage, alleviated the HP adhesion on cells and inhibited HP-caused inflammation. **(A)** The viability of cells was assessed following the treatment with varying concentrations of adenine and HP infection in GES1 cells for 12 hours. **(B)** The adhesion rate of HP to GES1 cells was measured by the urea test solution. **(C)** The levels of IL6 and TNF-α in the supernatants of RAW264.7 cells were quantified by ELISA. RAW264.7 cells were infected with HP and then treated with adenine. The level of statistical significance was represented by asterisks, with * or # indicating P < 0.05 and ** or ## indicating P < 0.01, respectively.

To observe the role of Adenine in HP infection, we detected the cell viability and HP adhesion on GES1 cells *in vitro*. The results indicated that adenine effectively restores the damage to GES1 cells caused by HP, and concurrently mitigates the adhesion of HP to the cells (**Figures 9A, B**). Furthermore, to determine whether adenine counteracts HP-induced inflammation, RAW264.7 cells were treated with both HP and adenine. The results indicated that HP infection significantly elevated the expression levels of IL6 and TNF-α proteins. Compared with the HP-only group, the expression of IL6 and TNF-α proteins was significantly decreased in the adenine-treated group (**Figure 9C**). The experimental data presented suggest that adenine exerts anti-inflammatory effects.

## Discussion

HP infection poses a significant risk factor and significantly contributes to a major public health concern, as well as the increasing prevalence of antimicrobial resistance (Dascalu et al., 2023). Current conventional therapies, primarily relying on antibiotics, face the dual hurdles of diminishing efficacy due to resistance and potential side effects, such as disrupting the gut microbiota and causing allergic reactions. In contrast, TCM offers a rich repository of potential solutions. Among TCM remedies, the helile formula, sourced from the “Tai ping sheng hui fang” with a specific 1:2 ratio of *Terminalia chebula* Retz. (10 g) and *Sanguisorba officinalis* L. (5 g), holds particular significance. In TCM theory, the combination of *Terminalia chebula Retz.* and *Sanguisorba officinalis L.* is based on the principles of balancing *yin* and *yang*, and regulating *qi* and *blood*. *Terminalia chebula* Retz., with its astringent property, is believed to restrain the abnormal movement of qi in the digestive tract, while *Sanguisorba officinalis* L., being cold in nature, clears heat and detoxifies, addressing the root cause of inflammation. This traditional wisdom aligns well with the modern understanding of HP-related gastrointestinal disorders, where symptoms such as diarrhea and dysentery often overlap with the inflammatory and infectious processes triggered by HP. However, despite its long-standing use in treating diarrhea and dysentery, the material basis and action mechanisms of the helile formula in the context of HP infection remain largely elusive. Thus, there is an urgent need for in-depth research to fully elucidate its potential value.

In this study, we identified the helile formula possess remarkable anti-HP infection properties. This discovery, stemming from the meticulous screening of ancient medicinal formulas, opens up a promising pathway for advancing innovative therapeutic approaches to manage HP infections. Furthermore, we have characterized the primary chemical constituents of helile formula, which include quinic acid, chebulic acid, terflavin B, gallic acid, terchebulin, 3,6-Digalloylglucose, punicalagin, chebulanin, 1,3,6-Trigalloylglucose, ellagic acid, chebulagic acid, chebulinic acid, and corilagin. Previous studies have demonstrated that ellagic acid possesses potent antimicrobial properties and inhibits the activity of Arylamine N-acetyltransferase (Chung, 1998). However, we found that the content of ellagic acid in helile formula accounts for 2.14%, indicating that the antibacterial active components in the helile formula may extend beyond ellagic acid. Recent research proved that the metabolic products of ellagic acid, urolithin A and urolithin B, exhibit beneficial effects against HP infection (Yu et al., 2022; Yu et al., 2023). Moreover, recent investigations suggest that corilagin (Zhang et al., 2013), chebulanin (Ou et al., 2024a), and 1,3,6-Trigalloylglucose (Ou et al., 2024b) possess anti-HP efficacy. Given the strong adaptability of HP and its resistance to antibiotics, it is nearly improbable to achieve complete inhibition of its growth through a single ingredient. This suggests that the potent antibacterial activity of helile formula likely results from a synergistic interaction among its multiple constituents.

To explore the inhibitory mechanism of the helile formula on HP, we designed and executed a series of *in vitro* and *in vivo* experiments. These results clearly showed that helile formula exhibited a significant inhibitory effect on HP growth and reduced its colonization on gastric mucosa. Through in-depth mechanism analysis, we found that the antibacterial effect of the helile formula was achieved through a synergistic action of multiple mechanisms, including inhibition of HP pathogenic factor secretion, reduction of its adhesiveness, disruption of cell structure, and enhancement of outer membrane permeability. Furthermore, the multi-component, multi-target treatment strategy employed by the helile formula not only improved treatment efficiency but also significantly reduced the risk of bacterial resistance development. This strategy provides a solid scientific basis for clinical treatment and highlights the crucial role of TCM in developing new HP treatment options. Based on these findings, we strongly recommend further scientific research on TCM to fully explore their potential in the treatment of HP infections.

Our study also provides compelling evidence that helile formula exhibits potent anti-inflammatory properties. Specifically, helile formula not only reinstates the protein expression levels suppressed by HP but also curbs the overproduction of inflammatory cytokines, including IL-6, TNF-α, IL-1β, TGF-β, and IFN-γ. HP initiates infection by attaching to the gastric epithelial cell surface, which triggers an inflammatory response. Multiple research indicates that certain compounds have the capacity to mitigate inflammatory responses and alleviate discomfort, such as quinic acid (Jang et al., 2017), gallic acid (Luan et al., 2023), punicalagin (Zoofeen et al., 2024), chebulanin (Liu et al., 2020), ellagic acid (Mazrooei et al., 2023), chebulagic acid (Shanmuganathan and Angayarkanni, 2018), and corilagin (Widowati et al., 2021). Based on these findings, the helile formula has the potential to emerge as a novel anti-inflammatory agent, offering an effective treatment option for HP-associated diseases.

Notably, emerging evidence suggests that the antibacterial and anti - inflammatory effects of the helile formula may be related to its modulation of the gut microbiota. HP infection and antibiotics treatment can disrupt the balance of the gut microbiota, but helile formula therapy can restore the gastrointestinal microbial community, leading to an increase in the abundance of beneficial bacteria such as *Muribaculaceae*. Nevertheless, our study has notable limitations, primarily due to the inability to isolate the specific bacterial strain for conducting direct interaction experiments. This constraint impedes the empirical validation of the hypothesized interactions.

Moreover, one of the most exciting findings of our study is that the helile formula exhibits antibacterial activity against HP through the modulation of specific metabolites. This may represent an additional mechanism by which helile formula functions to exert both antibacterial and anti-inflammatory effects. Interestingly, our metabolic pathway analysis has revealed that helile formula likely operates by regulating multiple metabolic pathways to manifest its antibacterial properties. These pathways may include amino acid metabolism, energy metabolism, lipid metabolism, and nucleotide metabolism. Based on metabolic pathway analysis, we hypothesize that helile formula’s primary antibacterial mechanism is through the regulation of specific metabolite levels, thereby affecting HP’s metabolic balance and leading to growth inhibition or death. These metabolites include certain amino acids, fatty acids, carbohydrates, or nucleotides. Helile formula, by modulating the metabolites of HP, may potentially influence critical biological processes such as energy metabolism and cell wall synthesis. Additionally, helile formula may also further impact the biological processes of HP and the host’s immune response by regulating other key metabolites such as adenine, panaxytriol, and 4-hydroxyglutamate semialdehyde. Our observations have shown a strong correlation between adenine and the enhancement of cellular damage, adhesion, and inflammation induced by HP, highlighting the critical role of adenine in these pathological processes.

In our comprehensive investigation of the helile formula’s antibacterial mechanism, we focused on its inhibitory effects on specific bacterial strains and its influence on the host immune response. Due to current experimental limitations, we were unable to conduct experiments using specifically altered single metabolites. In the future, we plan to conduct more in-depth studies to examine the impact of single-metabolite changes on the formula’s antibacterial effects. This will not only clarify the mechanism of action but also potentially lead to the development of more targeted therapeutic strategies. For example, identifying the most crucial metabolites involved in the formula’s antibacterial activity could enable the development of synthetic analogs or modified formulations for enhanced efficacy.

## Conclusion

In conclusion, our study has provided strong evidence that helile formula demonstrates potent antibacterial activity against HP both *in vitro* and *in vivo*. *In vitro*, helile formula increased bacterial outer membrane permeability, disrupted HP structure, inhibited toxin genes, and suppressed cell adhesion. *In vivo*, helile formula significantly reversed the activity of inflammatory factors induced by HP infection, such as IL-6, IL-1ß, TNF-α, TGF-β, and IFN-γ. Furthermore, our findings highlight the crucial role of helile formula in modulating alterations in gut microbiota induced by HP, particularly impacting families like *Muribaculaceae*, *Lachnospiraceae*, and *Lactobacilaceae*. Additionally, helile formula exerts anti-inflammatory effects against HP-induced injury by modulating key differential metabolites, such as N-alpha-Acetyl-L-lysine, adenine, 4-Hydroxyglutamate semialdehyde, N-Fructosyl pyroglutamate. Importantly, adenine recovered the HP-induced GES1 damage, alleviated the HP adhesion on cells, and suppressed the HP-induced IL6 and TNF-α. These findings underscore the potential of helile formula as a promising therapeutic agent for treating HP infections. Further exploration of the underlying mechanisms and clinical applications of helile formula is crucial to fully capitalize on its therapeutic potential.

## Data Availability

The data of 16S rDNA sequencing have been deposited into CNSA with accession number CNP0007554. The data that support this study’s findings, including any relevant details needed to reproduce the published results, are available from the corresponding author upon reasonable request.
